# Hepatitis E Virus Antibodies in Blood Donors, France

**DOI:** 10.3201/eid1712.110371

**Published:** 2011-12

**Authors:** Jean-Michel Mansuy, Richard Bendall, Florence Legrand-Abravanel, Karine Sauné, Marcel Miédouge, Vic Ellis, Henri Rech, François Destruel, Nassim Kamar, Harry R. Dalton, Jacques Izopet

**Affiliations:** Hôpital Purpan, Toulouse, France (J.-M. Mansuy, F. Legrand-Abravanel, K. Sauné, M. Miédouge, J. Izopet);; Royal Cornwall Hospital Trust, Truro, UK (R. Bendall, V. Ellis, H.R. Dalton);; Institut National de la Santé et de la Recherche Médicale, Toulouse (F. Legrand-Abravanel, K. Sauné, N. Kamar, J. Izopet);; Université Toulouse III Paul Sabatier, Toulouse (K. Sauné, N. Kamar, J. Izopet);; Etablissement Français du Sang Pyrénées-Méditerranée, Toulouse (H. Rech, F. Destruel);; Peninsula College of Medicine and Dentistry, Truro (H.R. Dalton);; Hôpital Rangueil, Toulouse (N. Kamar)

**Keywords:** viruses, zoonoses, foodborne infections, France, HEV, seroprevalence, blood donors, hepatitis E

## Abstract

Using a validated sensitive assay, we found hepatitis E virus (HEV) IgG in 52.5% of voluntary blood donors in southwestern France. This finding suggests HEV is highly endemic to this region. The high HEV prevalence may reflect local dietary practices, such as eating uncooked pork and game products.

It is now recognized that hepatitis E virus infection is not confined to developing countries. HEV infection is a growing public health concern in industrialized countries where the disease is mainly autochthonous, caused by HEV genotypes 3 (Europe) and 4 (People’s Republic of China and Japan), and is thought to be zoonotic (*1*).

In a previous study, we estimated that 16.6% of blood donors in the Midi-Pyrénées region of southwestern France have HEV antibodies (*2*). This rate was much higher than that measured in northern France (*3*), which suggests differences between these 2 populations and their exposure to HEV that we wished to explore further. However, it is difficult to make wider comparisons with seroprevalence studies from other areas because the various assays used differed in sensitivity and specificity (*4*). Because a recent study suggested that the HEV IgG assay we used in our original study lacks sensitivity (*5*), we repeated and extended the study using a more sensitive assay that has been validated by using serum from PCR-proven HEV genotype 3 infections (*5*).

## The Study

During September 2003 through May 2004, serum samples were collected from 512 adult blood donors 18–64 years old (median 42 years) and 188 children 2–4 years old. The blood donors were unpaid voluntary donors; the children were hospitalized in Toulouse for surgery or trauma. All were residents of the Midi-Pyrénées region. The prevalence of HEV IgG was determined by using the Wantai HEV IgG enzyme immunoassay (Wantai Biologic Pharmacy Enterprise, Beijing, People’s Republic of China), according to the manufacturer’s instructions. Details of baseline demographic data and putative risk factors were collected from blood donors by using a structured questionnaire. In addition, to assess the risk for foodborne infection, we tested 18 local pig-liver sausages for HEV RNA using a quantitative real-time PCR based on the open reading frame 2 region of the HEV genome (*6*).

**Figure 1:**
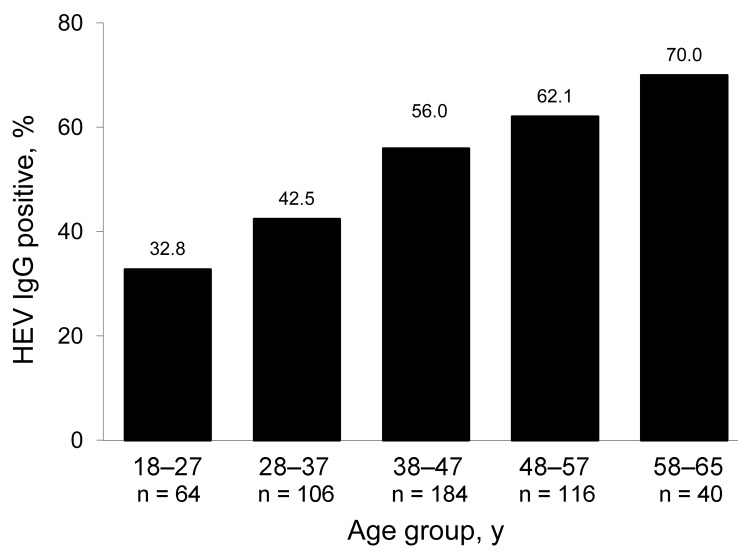
Prevalence of hepatitis E virus (HEV) IgG in 512 blood donors by age group, Midi-Pyrénées region, France, 2003–2004.

**Figure 2:**
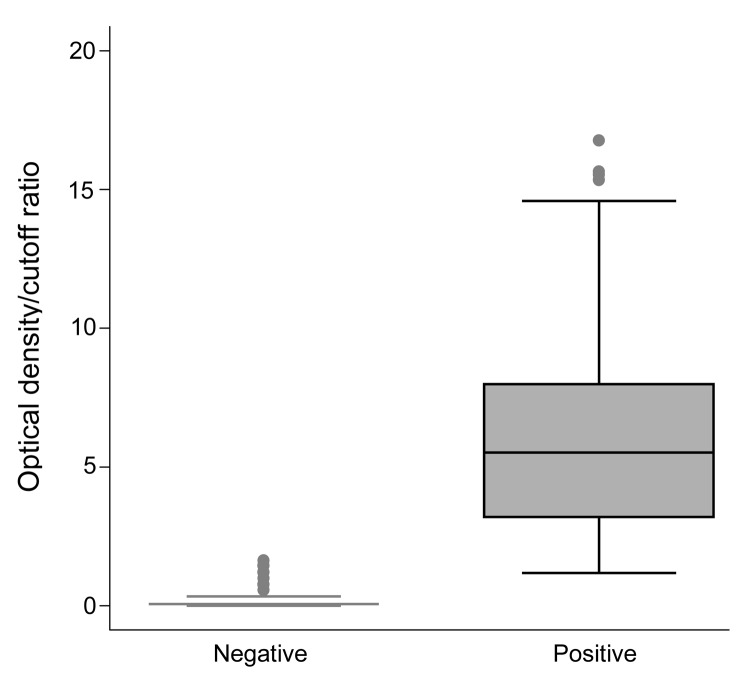
Distribution of optical density/cut off ratios for hepatitis E virus IgG in positive and negative samples from 512 blood donors, Midi-Pyrénées region, France, 2003–2004. Whiskers represent percentiles.

HEV IgG was detected in 268 (52.5%) of 512 (95% confidence interval [CI] 48.2%–56.8%) of the blood donors. Seroprevalence increased with age (Figure 1). The ranges of optical density/cutoff ratios for positive and negative samples showed a clear bimodal distribution (Figure 2). Of 244 rural donors, 63.1% (95% CI 57%–69.2%) were anti-HEV positive compared with 42.9% (95% CI 37–48.8) of 268 urban donors (p<0.01). For children, seroprevalence was 3.7% (95% CI 1.0%–6.5%). The mean ± SD optical density/cutoff ratio of the positive samples was 5.43 ± 3.93 for children and 5.99 ± 3.52 for adults. Although several factors were associated with the presence of HEV IgG after univariate analysis, multivariate analysis identified only age, rural residence, hunting, and contact with cats as factors independently associated with HEV IgG positivity (Table 1).

**Table 1:** Prevalence of HEV IgG, demographics, and potential risk factors for 512 blood donors, Midi-Pyrénées region, France, 2003–2004*

Analysis and risk factor	% Donors with HEV IgG	Odds ratio (95% CI)	p value
Univariate analysis			
Age		1.24 (1.14–1.35)	<0.001
Sex, M/F	51/55	1.14 (0.79–1.64)	NS
Rural residence	59	2.27 (1.60–3.25)	<0.001
Gardening	61	1.75 (1.22–2.5)	<0.01
Kitchen gardening	69	2.23 (1.30–3.87)	<0.001
Hunting	80	3.82 (1.41–10.4)	<0.01
Contact with farm animals	57	1.6 (1.12–2.29)	<0.01
Contact with dogs	58	1.42 (0.99–2.0)	NS
Contact with cats	59	1.49 (0.04–2.13)	<0.05
Contact with horses	54	1.05 (0.35–3.17)	NS
Contact with pigs	50	0.89 (0.25–3.11)	NS
Contact with poultry	51	0.93 (0.49–1.80)	NS
Contact with wild animals	74	2.73 (1.20–6.23)	<0.05
Travel outside France	52	0.54 (0.35–0.83)	<0.01
Travel outside Europe	50	0.58 (0.38–0.89)	<0.05
Multivariate analysis			
Age		1.20 (1.10–1.31)	<0.01
Rural residence		1.80 (1.24–2.62)	<0.01
Hunting		4.11 (1.35–12.5)	<0.05

Contact with cats

1.6 (1.10–2.34)

<0.05

*HEV, hepatitis E virus; CI, confidence interval; NS, not significant.

HEV RNA was found in 8 (44%) of the 18 sausages tested by real-time PCR (Table 2). The virus load ranged from 100 (the limit of detection for this assay) to 668,520 copies/g. We attempted to genotype HEV RNA–positive samples by sequencing a 189-nt fragment of the open reading frame 2 gene (*7*). This was successful only for the sample with the highest virus load. The virus was identified as HEV genotype 3.

**Table 2:** Detection and quantification by real-time PCR of HEV RNA in pig-liver sausages purchased from markets in the Midi-Pyrénées region, France, 2003–2004*

Sample	Market	HEV RNA	HEV RNA concentration†	Genotype
1	A1	Negative		
2	A2	Negative		
3	A3	Negative		
4	A4	Positive	4,100	NA
5	A5	Positive	175	NA
6	A6	Positive	240	NA
7	A7	Negative		
8	B	Positive	100	NA
9	C	Negative		
10	D	Positive	8,200	NA
11	E	Negative		
12	F	Positive	668,520	3
13	G	Negative		
14	H	Negative		
15	I	Positive	120	NA
16	J	Negative		
17	K	Negative		
18	L	Positive	48,550	NA

*A1–A7 indicate different shops in the same market. HEV, hepatitis E virus; NA, no PCR amplification with primers used for genotyping. †Copies/g.

## Conclusions

We determined that the HEV IgG prevalence among blood donors in Midi-Pyrénées is 52.5%, the highest seroprevalence reported in an industrialized country. This rate is 3.1 times higher than our previous estimate (16.6%) for the same population (*2*). The implication is that HEV is hyperendemic to Midi-Pyrénées.

Although surprising, we believe these results are valid for several reasons. First, the Wantai assay used to assess HEV seroprevalence has been validated for this purpose in the United Kingdom, another region where HEV-3 predominates (*5*). The greater proportion of reactive serum seen with this assay is unlikely to have resulted from nonspecific reactivity because the assay produced a clear distinction between negative and positive samples, and only a small proportion of young children tested positive. Our findings agree with those of another study that found a much increased HEV seroprevalence when the more accurate test was used (*5*). Second, autochthonous HEV genotype 3 hepatitis in Midi-Pyrénées is common. The estimated rate of acquisition of HEV infection in organ transplant recipients in Toulouse is 3.2 per 100 person-years (*8*). This figure is derived from regular monitoring by using sensitive molecular techniques and does not depend on serologic assays. As noted in other countries (*9**,**10*), the percentage of HEV-positive serum increased with age, which is consistent with cumulative exposure to infection over time.

HEV is usually transmitted orally, and foodborne transmission of zoonotic strains has been demonstrated. Hunting of wild boar and deer is popular in Midi-Pyrénées, particularly in rural areas. Both species have been identified as sources of human infection (*11*). The consumption of uncooked game meat, which is traditional in this area, could explain the high HEV antibody prevalence in 20 (80%) of 25 hunters. Further evidence comes from a case–control study among organ transplant recipients in Midi-Pyrénées that demonstrated that the only factor independently associated with HEV infection was consumption of game meat (*6*). Some of these foods have been shown to contain HEV RNA, and phylogenetic analysis demonstrated that these strains were closely related to human strains (*8*).

Another suspected zoonotic source of HEV genotype 3 infection is the domestic pig (*12*). Hepatitis E cases have been linked to eating uncooked pork-liver sausage in southeastern France (*13*). We found that a high proportion (44%) of pig-liver sausages purchased in Toulouse contained HEV RNA. These air-dried sausages are popular in Midi-Pyrénées and are usually eaten raw. Their infectivity is unknown, but cell-culture experiments have demonstrated that high virus loads correlate with high infectivity (*14*).

In addition to direct foodborne transmission, the growing boar population in Midi-Pyrénées and the spreading of pig manure on land may pose indirect risks through fecal contamination of soil and watercourses. These 2 factors and the 2 foodborne sources of HEV might explain the high HEV antibody prevalence in Midi-Pyrénées and the statistical association of HEV seropositivity with rural residence and hunting, but they cannot explain the association with cat contact. HEV RNA has not yet been detected in domestic cats.

The high percentage of donors with HEV antibodies in our area contrasts with the low recorded incidence of autochthonous hepatitis E in France and other industrialized countries (*1**,**15*). Even though we documented dozens of hepatitis E cases in Midi-Pyrénées during the past decade, the remarkably high seroprevalence in this area suggests that most infections must be subclinical or unrecognized. However, in susceptible persons, such as organ transplant recipients and patients with chronic liver disease, the consequences of HEV infection are grave and raise public health, as well as food and environmental safety, concerns.

HEV is highly endemic to the Midi-Pyrénées region in southwestern France. We showed that seroprevalence increases with age and is associated with rural residence, hunting, and exposure to cats. These associations became apparent only when we used a sensitive assay to detect HEV IgG. The reasons for the high HEV prevalence in this population are uncertain but may be due, at least partially, to the culinary culture of the local community. Thorough cooking of game meat and pork products would help minimize the risk for HEV infection and could form part of a public health initiative in this area.
